# Cytotoxicity and Effects on the Synapsis Induced by Pure Cylindrospermopsin in an E17 Embryonic Murine Primary Neuronal Culture in a Concentration- and Time-Dependent Manner

**DOI:** 10.3390/toxins14030175

**Published:** 2022-02-26

**Authors:** María G. Hinojosa, Ana I. Prieto, Clara Muñoz-Castro, María V. Sánchez-Mico, Javier Vitorica, Ana M. Cameán, Ángeles Jos

**Affiliations:** 1 Area of Toxicology, Faculty of Pharmacy, University of Sevilla, C/Profesor García González 2, 41012 Sevilla, Spain; mhinojosa1@us.es (M.G.H.); camean@us.es (A.M.C.); angelesjos@us.es (Á.J.); 2 Department of Biochemistry and Molecular Biology, Faculty of Pharmacy, University of Sevilla, 41012 Sevilla, Spain; cmunozcastro@mgh.harvard.edu (C.M.-C.); marsanmic@gmail.com (M.V.S.-M.); vitorica@us.es (J.V.); 3 Biomedicine Institute of Sevilla (IBiS), Universitary Hospital Virgen del Rocío/CSIC/University of Sevilla, 41013 Sevilla, Spain; 4 Biomedical Research Center in Network on Neurodegenerative Diseases (CIBERNED), 28031 Madrid, Spain

**Keywords:** cylindrospermopsin, cytotoxicity, primary neuronal cultures, neurotoxicity, synapsis

## Abstract

Cylindrospermopsin (CYN) is a cyanotoxin whose incidence has been increasing in the last decades. Due to its capacity to exert damage at different levels of the organism, it is considered a cytotoxin. Although the main target organ is the liver, recent studies indicate that CYN has potential toxic effects on the nervous system, both in vitro and in vivo. Thus, the aim of the present work was to study the effects of this cyanotoxin on neuronal viability and synaptic integrity in murine primary cultures of neurons exposed to environmentally relevant concentrations (0–1 µg/mL CYN) for 12, 24, and 48 h. The results demonstrate a concentration- and time-dependent decrease in cell viability; no cytotoxicity was detected after exposure to the cyanotoxin for 12 h, while all of the concentrations assayed decreased this parameter after 48 h. Furthermore, CYN was also demonstrated to exert damage at the synaptic level in a murine primary neuronal culture in a concentration- and time-dependent manner. These data highlight the importance of studying the neurotoxic properties of this cyanotoxin in different experimental models.

## 1. Introduction

Cylindrospermopsin (CYN) is a secondary metabolite produced by several species of cyanobacteria, such as *Cylindrospermopsis raciborskii*, *Aphanizomenon ovalisporum*, *Raphidiopsis curvata*, *Chrysosporum ovalisporum*, *Lyngbia wollei*, *Anabaena bergii*, *Umezakia natans*, *Oscillatoria* sp., etc. [1,2]. Structurally, this cyanotoxin consists of a tricyclic guanidine moiety with a hydroxymethyl uracil [3]. Due to its zwitterionic nature and its low molecular weight, this molecule is highly soluble in water and stable in many environmental conditions [4,5]. These factors, together with the cosmopolitan distribution of producing species, have led to an increase in its incidence in the last decades [6]. In this context, Yang et al. [7] reported the presence of CYN in water bodies on six continents, and it was found to be quite common in waters in Europe, Asia, Oceania, and North America and less documented in South America and Africa. Of the tested samples, the highest value reported was 1050 µg/L CYN in an Australian water supply [7].

Furthermore, cyanotoxins have demonstrated their capacity to bioaccumulate in several organisms in different parts of the food chain [8,9], such as mollusks [10], fish [11], toads, and plants [5,12]. Thus, their main route of exposure would be the oral route through contaminated water or food intake. However, dermal, inhalation, and parenteral exposures are also possible. The most famous case of human intoxication caused by CYN was an outbreak of hepatic enteritis on Palm Island, Queensland, Australia, where over 150 people presented symptoms of headache, anorexia, vomiting, hepatomegaly, bloody diarrhea, and dehydration [13,14]. All of them reported water intake from a reservoir that contained a bloom of a CYN-producing strain of *R. raciborskii* [13]. Furthermore, in Brazil, CYN was also detected in Caruaru syndrome, caused by the presence of a different cyanotoxin, microcystin, in the water from a hemodialysis clinic, although no CYN quantification was performed, so it was not possible to evaluate its contribution to the effects observed [6,15]. Cylindrospermopsin is classified as a cytotoxin and is able to exert toxicity in many different cell lines, such as HepG2, Caco-2, and CHO, among others [7,16,17,18]. Moreover, many cases of animal intoxication have been reported, most of them leading to death, demonstrating CYN bioaccumulation mainly in the liver, although other organs were also affected [7]. In this sense, this cyanotoxin has been demonstrated to exert damage in different organs, such as the lungs, heart, kidneys, thymus, spleen, and nervous system [7,19,20].

Different in vivo laboratory studies have shown that CYN produces oxidative stress and histopathological lesions in the liver, kidney, heart, intestines, and gills in tilapia that were subchronically exposed (14 days) by immersion to lyophilized *Aphanizomenon ovalisporum* cells at environmentally relevant concentrations [21]. Moreover, CYN induced significant inhibition of AChE activity and increased LPO levels, as well as relevant histopathological alterations in the brain of fish subchronically exposed to the toxin [22]. Thus, CYN has been demonstrated to be able to cross the blood–brain barrier, as its presence has been detected in the brain of at least three different fish species [22,23,24]. In mice that were subchronically orally exposed to purified CYN (75–300 μg/kg/d) for 90 days, toxicity was noted at all dose levels tested [25]. Thus, Chernoff et al. [25] observed elevated organ-to-body weight ratios of the liver and kidney, important histopathological lesions (hepatocellular hypertrophy, cord disruption in the liver, and renal cellular hypertrophy), and alterations of some biochemical parameters. In contrast, recently, Diez-Quijada et al. [26] carried out a study in rats with repeated oral exposure to pure CYN (up to 75.0 μg/kg bw) for 28 days, and they did not find statistically significant differences in hematological and biochemical parameters.

The main mechanism of action of CYN is protein synthesis inhibition, including the synthesis of the tripeptide glutathione (GSH), which leads to oxidative stress, which, in turn, leads to increased lipid peroxidation, DNA damage, and apoptosis [7,20,24,27,28,29,30]. However, the neurotoxic effects of this cyanotoxin have been poorly studied so far, with previous reports mainly demonstrating apoptosis, oxidative stress, and alteration of acetylcholinesterase activity, but no studies have focused on neuronal function [19,31]. Thus, the objective of the present work was to assess the neurotoxic effect of CYN on cell viability and synaptic density in murine primary cultured neurons in order to predict a possible mechanism involved in this neurotoxicity.

## 2. Results

### 2.1. Cytotoxicity of CYN in Murine Primary Neuronal Culture

**Figure 1:**
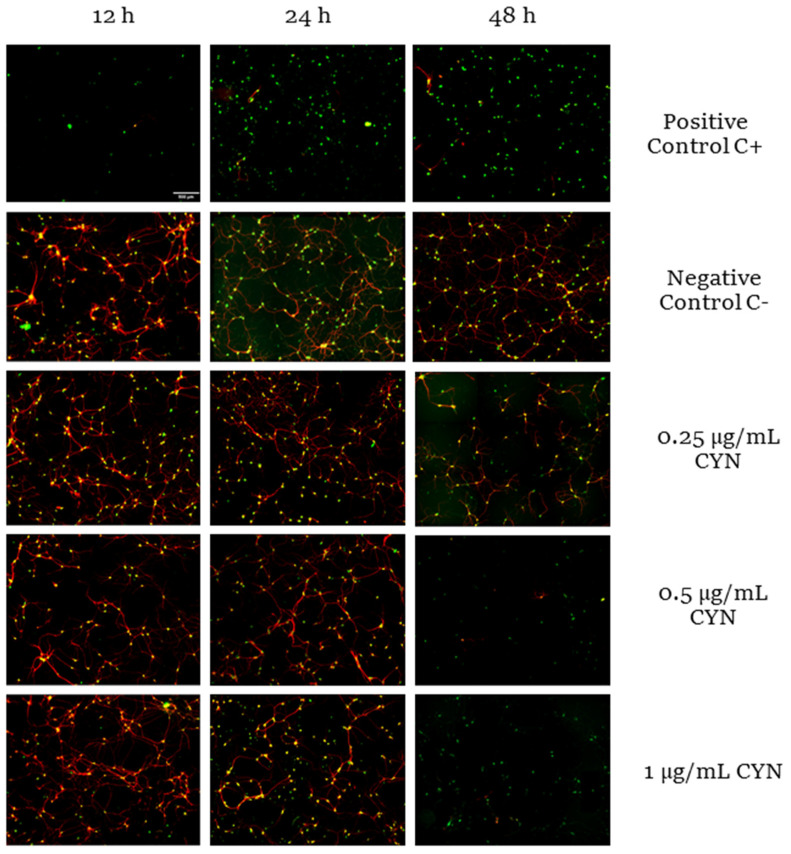
Images of murine primary neurons from E17 embryonic mice after exposure to 0.25, 0.5, and 1 µg/mL for 12, 24 and 48 h taken with the Apotome Zeiss epifluorescence microscope, where NeuN and MAP2 are shown in green and red, respectively. Control C−: neurobasal medium with B27 supplements were used as a negative control. Control C+: neurobasal medium without B27 were used as a positive control of reduced neuronal viability. Scale bars, 500 μm.

**Figure 2:**
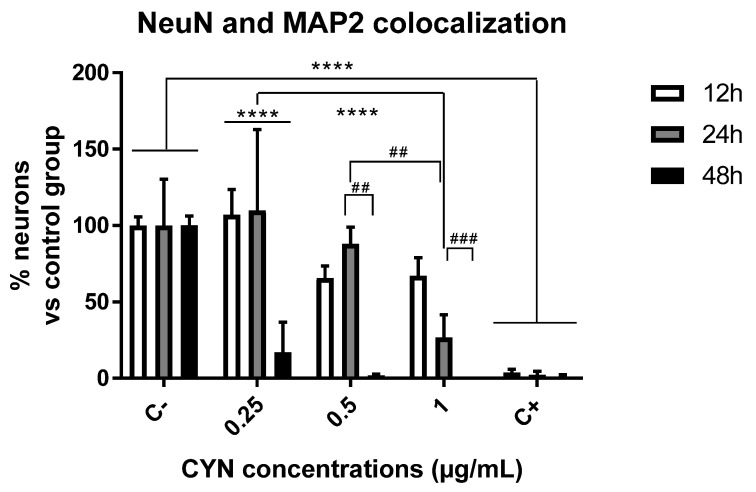
Effect of 0–1 µg/mL CYN on neuronal viability quantified by NeuN and MAP-2 colocalization immunocytochemistry in E17 primary cultures exposed to the toxin for 12, 24, and 48 h (*n* = 4). **** *p* < 0.0001, compared to their respective control group; ## *p* < 0.005, ### *p* < 0.0005, compared to the same concentration of exposure. Control C−: neurobasal medium with B27 supplements were used as a negative control. Control C+: neurobasal medium without B27 were used as a positive control of reduced neuronal viability.

Neuronal viability was measured by immunocytochemistry using the markers NeuN and microtubule-associated protein 2 (MAP-2). NeuN is a specific protein from postmitotic neurons used to assess neuronal differentiation [32]. In addition, MAP-2, present in postmitotic neurons, is a structural protein necessary to maintain neuroarchitecture and plays a role in responses to growth factors, neurotransmitters, synaptic activity, and neurotoxins [33,34]. Thus, both markers were used not only to study viability but also to confirm that the cells under study were specifically neurons. For this purpose, primary neuronal cultures were exposed to different concentrations of CYN (0.25–1 µg/mL) for different durations (12, 24, and 48 h), and the number of neurons was quantified by MAP2 and NeuN immunocytochemistry. As shown in Figure 1, a significant decrease in neuronal viability was observed after 48 h of exposure for all concentrations of CYN assayed and after 24 h of exposure only for the highest concentration (1 µg/mL CYN). The treatment of neurons without the B27 supplement served as a positive control of neuronal death because it is an essential supplement for the survival of primary neurons in vitro. Ultimately, important differences were observed in the cytotoxicity of CYN in murine primary neurons in a time- and concentration-dependent manner (Figure 2).

### 2.2. Effect of CYN on the Number of Synapses in Murine Primary Neuronal Culture

**Figure 3:**
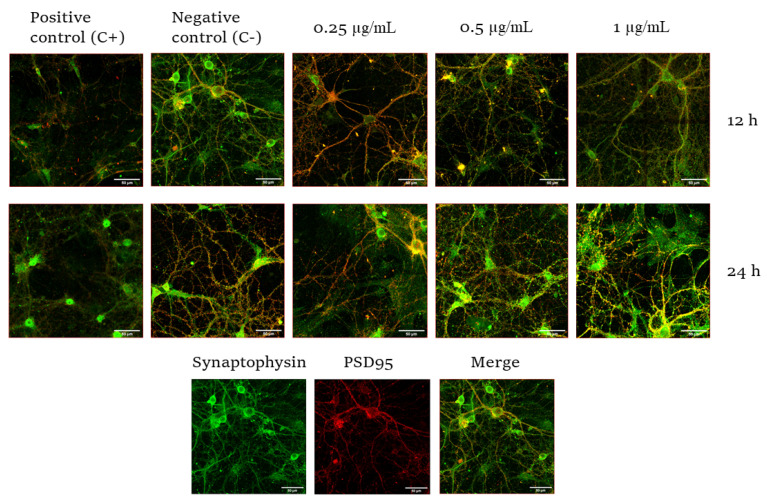
Representative images of murine primary neurons (synaptophysin, green; PSD95, red) from E17 embryonic mice after exposure to 0.25, 0.5, and 1 µg/mL for 12 and 24 h taken with the ZEISS LSM 710 confocal microscope. Control C−: neurobasal medium with B27 supplements were used as a negative control. Control C+: neurobasal medium without B27 were used as a positive control of reduced neuronal viability. Scale bars, 50 µm. Merging of pre- and postsynaptic markers.

**Figure 4:**
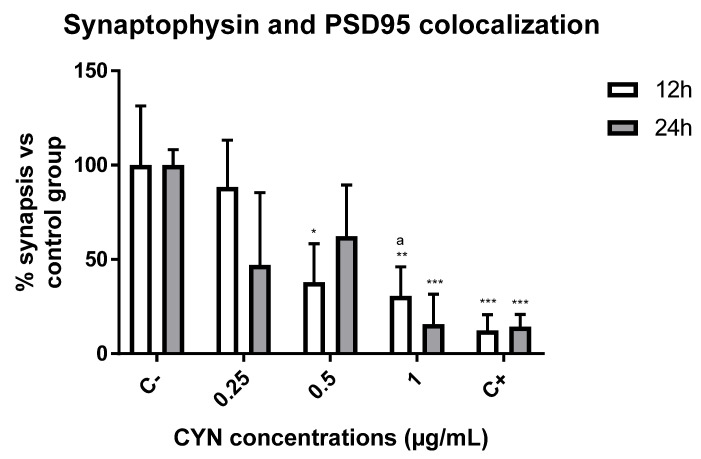
Effect of 0–1 µg/mL CYN on synaptic integrity measured by synaptophysin and PSD95 colocalization in E17 primary neuronal cultures for 12 and 24 h (*n* = 4). * *p* < 0.05, ** *p* < 0.005, *** *p* < 0.0005, compared to their respective negative control. a *p* < 0.05, compared to 0.25 µg/mL at the indicated time of exposure. Control C−: neurobasal medium with B27 supplements were used as a negative control. Control C+: neurobasal medium without B27 were used as a positive control of reduced neuronal viability.

The number of synapses was measured by studying synaptophysin 1 and PSD95 colocalization. Synaptophysin is an integral membrane protein of small synaptic vesicles and is involved in the exocytosis of stored neurotransmitters [35]. In addition, PSD95 is one of the most abundant proteins in postsynaptic neurons and is involved in synaptic maturation [36]. The colocalization of both proteins allows the detection and quantification of synapses. In order to study the effect of CYN on synaptic integrity, primary neuronal cultures were exposed to different concentrations of this toxin for different durations, and the colocalization of synaptophysin and PSD95 was used to quantify the number of synaptic connections. The results showed that the highest decrease in synaptic number occurred at a concentration of 1 µg/mL CYN in murine primary neurons. In fact, significant concentration-dependent differences (0.25 vs. 1) in this neuronal function were observed in cells exposed to the toxin for 12 h (Figure 3 and Figure 4). The treatment of neurons without the B27 supplement induced the loss or de-colocalization of these two synaptic markers, so it served as a positive control of reduced synaptic integrity.

## 3. Discussion

Neurodegenerative diseases are a leading cause of death and affect a huge number of people worldwide [37]. Moreover, it is known that some toxicants may cause or/and hasten neuronal senescence [38]. Currently, studies concerning the neurotoxic effects of CYN are very scarce. CYN is known to be cytotoxic, and this has been proven in different cell lines derived from the nervous system used to study its effects, such as SH-SY5Y cells, BV-2 cells, N2a cells, etc. [19,39]. In vitro cellular models are easier to use than in vivo experimental models [40]. In addition, studies performed using cells are very reproducible and reliable, whilst being time-saving and cost-effective, and provide a better-controlled setting. In this regard, primary neuronal cultures and neuronal cell lines derived from rodents represent a useful tool to study the potential neurotoxicity of chemicals [41]. Moreover, primary neuronal cell cultures can provide a more similar phenotype to the real situation than immortal cell lines since these may acquire genetic instability during long times of passage, and neurites may not represent true axons or dendrites [41]. Specifically, the hippocampus is an area of the brain that possesses remarkable neuronal activity, and it is characterized by changes in synaptic responsiveness [42]. Recently, primary cultures of this brain area have been used to investigate aspects related to neurodegenerative diseases, such as the effect produced by toxic aggregates of alpha-synuclein [41], the antioxidant and neuroprotective effects of zolpidem [43], or the protective effects of cannabinoid receptor 2 agonist compounds against neuronal toxicity induced by the accumulation of beta-amyloid peptides [44]. In all of these studies, E17 embryonic murine primary hippocampal neuronal culture was selected in order to study the effects of CYN in neurons that are more similar to human neurons. In this regard, the markers NeuN and MAP-2 were used to measure cytotoxicity and also to confirm their maturity and neuronal nature; these markers are employed in the study of different neurodegenerative diseases, such as Parkinson’s or Alzheimer’s [45,46].

Taking these facts into account, CYN was demonstrated, for the first time, to cause a concentration- and time-dependent decrease in viability in a range of 0–1 µg/mL after 24 and 48 h of exposure in our experiment, causing significant changes compared to the respective negative control group. At the lowest concentrations tested in our experimental model (0.25 and 0.5 µg/mL), cytotoxicity was only observed after 48 h of exposure. However, the highest CYN concentration (1 µg/mL) was demonstrated to cause a significant decrease in neuronal viability after 24 and 48 h. This finding is important, as the concentration range used in this study has been naturally found in environmental samples [7]. This is the first study to use neuron-specific biomarkers such as NeuN and MAP2 to assess CYN cytotoxicity. These results are in agreement with those obtained by Hinojosa et al. [31] in the undifferentiated human neuroblastoma cell line SH-SY5Y, where the EC50 reported was around 1 µg/mL after 24 h. However, when the same authors studied its effects on differentiated SH-SY5Y cells, which are supposed to be more similar to primary cultures, the EC50 value obtained after 24 h of exposure was 0.3 µg/mL, with these cells being the most sensitive to the cyanotoxin. This might be due to differences between the biomarkers used to measure cytotoxicity in the two studies: the MTS assay in Hinojosa et al. [31] compared to NeuN and MAP2 biomarkers in the present work. Thus, the EC50 values were higher when these authors studied viability by performing the neutral red uptake assay or the protein content assay, demonstrating the sensitivity of the assay to those experimental conditions.

Nonetheless, in the present study, viability was studied by using specific biomarkers from mature neurons that are supposed to be present in primary neuronal cultures in order to not only assess the effects of the cyanotoxin on the cell viability but also confirm that our culture contains mainly neurons after isolation from the hippocampus [47]. The concentration- and time-dependent decrease observed in the present study is also in agreement with the results presented by Takser et al. [48], who exposed N2a murine neuroblastoma-derived cells and BV-2 microglia murine cells to 0.1 and 10 µM CYN and observed a significant decrease in cell viability after exposure for 24, 48, and 72 h to both concentrations. Although they just studied two concentrations, and thus, it is difficult to compare the responses to the ones obtained in our study, the BV-2 cells were demonstrated to be the most sensitive cell line in their study, showing more significant changes after both times of exposure. This demonstrates that the behavior of the cells can differ according to the experimental model, but a concentration- and time-dependent trend was observed in all of them. In addition, both Takser et al. [48] and Hinojosa et al. [31] demonstrated apoptosis in their respective cell models.

**Figure 5:**
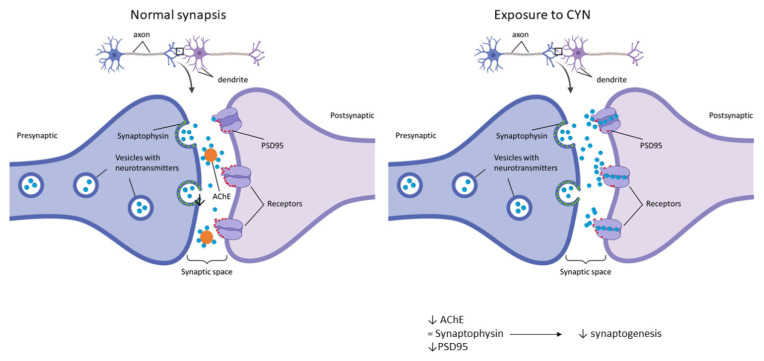
Schematic diagram of normal synapsis vs. synapsis after exposure to CYN.

Concerning neurofunctional studies, in our model, the markers synaptophysin and PSD95 were employed to study the number of synapses through their colocalization. These markers are usually employed to study the effects of a toxin during synaptic development or to study synaptogenesis during development [46,49]. In this study, CYN caused significant decreases in these synaptic markers after exposure to 0.5 µg CYN/mL for 12 h in our experimental model and after exposure to 1 µg CYN/mL for both exposure times. To our knowledge, this is the first study focused on synaptic response disruption caused by CYN. The only reports studying the effects of CYN on synaptic function have been carried out indirectly by studying acetylcholinesterase activity (AChE), as a decrease in this enzyme would lead to an overstimulation of postsynaptic neurons and, thus, lead to cholinergic disturbances. In this regard, the only study performed in vitro was the one carried out by Hinojosa et al. [31], who observed no alteration in undifferentiated SH-SY5Y cells after exposure to 0–1 µg/mL CYN for 24 h, while in differentiated cells, 0.15 and 0.3 µg/mL caused a decrease in this enzymatic activity. This is in agreement with our results, as the present study also demonstrated a significant decrease in synaptic integrity after 12 h of exposure. The disruption of normal synaptic formation and function is often seen as an endpoint in neurological diseases. Moreover, the ability to quantify changes in structural synaptic formation after treatment with a toxicant may not only provide much-needed information about the extent of the effect but may also provide clues to the underlying mechanisms [42]. It is also worth mentioning that CYN also caused a decrease in AChE in vivo in tilapia fish exposed to a CYN-producing strain of *C. ovalisporum* for 14 days [22]. The decrease in AChE could be due to a link between CYN and AChE, which would lead to the accumulation of ACh in the receptors and, thus, overstimulation, which is in agreement with the results obtained in our study. In this regard, as observed in Figure 5, the number of presynaptic markers did not vary after exposure to CYN, while the quantity of the red signal decreased in our study. Taking into account that PSD95 is present in postsynaptic excitatory neurons, the overstimulation of those neurons by the lack of AChE would lead to a decrease in this signal, decreasing synaptogenesis. This decrease could result in the generation of neurodegenerative diseases or problems during neurodevelopment in the case of exposure during pregnancy [50]. In addition, it is important to take into account that the primary cultures were obtained from the hippocampus; a lack of synapsis at this level would lead to learning and memory impairment, together with changes in spatial navigation, emotional behavior, and regulation of hypothalamic functions [51].

The toxicity profile of CYN partially resembles that of microcystins in the sense that both of them are primarily hepatotoxic, but they have been reported to also induce potential neurotoxic effects [19]. Thus, the neurotoxicity of cyanotoxins in general and of CYN in particular is worthy of research and requires further attention. In this regard, the use of novel and advanced experimental models such as primary neuronal cultures could contribute to the progress of scientific knowledge on this topic.

## 4. Conclusions

Our findings demonstrate that CYN is able to cause effects on murine primary cultures of neurons, causing a concentration- and time-dependent decrease in viability after exposure to environmentally relevant concentrations of the toxin. In addition, for the first time, it is shown that CYN is able to cause a concentration-dependent decrease in the synaptic number. Thus, these results highlight the importance of carrying out more experiments concerning the neurotoxic properties that CYN can exert in order to properly evaluate its risk and its possible implication in neurodegenerative diseases.

## 5. Materials and Methods

### 5.1. Materials

Cylindrospermopsin (purity > 95% by HPLC) was purchased from Enzo Life Sciences (Barcelona, Spain). Dako Fluoromount™ aqueous mounting (F4680), glucose (2.5 M) (68769), HEPES (68769), HBSS 10× (H9269), L-cysteine (C7352-256), poly-D-lysine (P6407), Triton™ X-100 solution (93443), and trypsin inhibitor (T9128) were purchased from Sigma Aldrich (Madrid, Spain). PBS 10× (X0515-500), penicillin + streptomycin 100× (L0022-100), DMEM-F12 (L0090-500), glutamine (Q) 100× (X0550-100), and horse serum (5091H-500) were obtained from Biowest (Barcelona, Spain). Sodium pyruvate (11360-070), neurobasal (21103-049), B27 supplement 50× (17504-044), and Glutamax (35050061) were purchased from Gibco (Madrid, Spain). Papain (LS003127) was purchased from Worthington (Barcelona, Spain). Ethanol absolute (20821.330) was purchased from VWR Chemicals (Madrid, Spain). Paraformaldehyde 4% (15434389) was purchased from Alfa Aesar™(Madrid, Spain). The antibodies anti-microtubule-associated protein 2 (MAP2) (AB5622), anti-postsynaptic density protein 95 (PSD95) (MAB 1596), and anti-NeuN clone A60 (NeuN) (MAB377) were obtained from EDM Millipore Corp (Darmstadt, Germany). Synaptophysin 1 (101002) was purchased from Synaptic Systems (Goettingen, Germany). Secondary antibodies Alexa Fluor™ 546 donkey anti-rabbit IgG (H+L) (A10040) and Alexa Fluor™ 488 donkey anti-mouse IgG (H+L) (A21202) were purchased from Invitrogen by Thermo Fisher Scientific (Madrid, Spain).

### 5.2. Toxin Test Solution

A stock solution of 1 mg/mL CYN was prepared in sterilized Milli-Q water and maintained at −20 °C until its use.

### 5.3. Animals

Pregnant mice (CD1) at 17 days of gestation were used to obtain hippocampal neurons. All animals received human care in accordance with the directive for the protection of animals utilized for scientific purposes: Directive 2010/63/UE, Decision 2020/569/UE, and RD 1386/2018. All methods were authorized by the Ethical Animal Experimentation Committee of the University of Sevilla and by the Junta de Andalucía (project no. 04/03/2019/020).

### 5.4. Primary Culture of Hippocampal Neurons and Toxin Exposure

Primary cultures were obtained from the hippocampus of E17 embryonic CD1 mice according to the protocol described by Zhao et al. [39]. Briefly, the hippocampus was isolated in cold dissociation medium with HBSS, glucose (10 mM), and HEPES (10 mM). Afterwards, they were incubated for 3 min at 37 °C with slow agitation in a solution of papain (41 U/mL) and L-cysteine 0.06% (p/v) in dissociation medium. Then, the samples were washed 3 times with trypsin inhibitor (1 mg/mL) in dissociation medium and then incubated again for 4 min at 37 °C with slow agitation. After that, the hippocampi were mechanically disaggregated in the presence of medium containing DMEM-F12 supplemented with glutamine (2 mM), sodium pyruvate (1%), glucose (20 mM), and inactivated horse serum (10%). Then, 80,000 cells/well were seeded in coverslips in 24-well plates previously coated with poly-D-lysine. After 2 h, the medium was replaced by neuronal medium containing neurobasal, B27 (2%), Glutamax (2 mM), and penicillin and streptomycin (50 µg/mL). One-quarter of the medium was refreshed every 2–3 days. After 21 days, the neurons were exposed to 0.25, 0.5, and 1 µg/mL CYN for 12, 24, and 48 h. These experimental concentrations were selected to encompass the range of environmentally relevant concentrations found in nature (up to 1 µg/mL).

### 5.5. Sample Fixation and Immunocytochemistry

The cells were fixed with paraformaldehyde (4%) for 20 min at room temperature. Then, PBS was used to wash the samples twice, and ethanol (70%) was added for maintenance at 4 °C until the performance of the immunocytochemical analysis. After PBS washing, the cells were incubated with 0.1% Triton-X 100 in PBS for 1 h at room temperature to permeabilize. Then, samples were incubated with blocking solution (0.1% Triton-X 100 and 1% bovine serum albumin (BSA) in PBS) for 3 h. After that, primary antibodies (NeuN 1:1000 and MAP2 1:1000, or synaptophysin 1 1:500 and PSD95 1:100) were added to a solution with 0.1% Triton-X 100 and 1% BSA, which was left at room temperature for 30 min and then incubated at 4 °C overnight, except for PSD95, which required incubation for 3 days. Finally, samples were washed with 0.1% Triton-X 100 in PBS and incubated with 1:500 dilution of the secondary antibodies (Alexa Fluor™ 546 donkey anti-rabbit IgG and 488 donkey anti-mouse IgG) in PBS with 0.1% Triton-X 100 and 1% BSA for an hour. Finally, the samples were washed twice with PBS, and the coverslips were added to the slides with Fluorescent Mounting Medium.

### 5.6. Image Acquisition and Analysis

The images were obtained by Zeiss apotome epifluorescence microscope at 20× to capture viability images and ZEISS LSM 710 confocal microscope using a 63× oil-immersion objective lens to capture neuronal synapsis images. Then, they were analyzed using ImageJ (FIJI) and the plugins “Cell Counter” to quantify the number of neurons and “NeuronJ” and “SynaptCountJ” to quantify PSD95 and synaptophysin colocalization in neuronal synapses [52].

### 5.7. Data Analysis

Data are presented as mean ± standard deviation (SD) in relation to control. Statistical analysis was carried out using analysis of variance (ANOVA), followed by Tukey’s multiple comparison tests using GraphPad Prism 7.05.
